# Caregiving During COVID-19: A Multi-State Qualitative Study of Family Caregiver Experiences and Decision Making

**DOI:** 10.1093/geroni/igab046.1702

**Published:** 2021-12-17

**Authors:** Tonie Sadler, Kevin Yan, Daniel Brauner, Harold Pollack, R Tamara Konetzka

**Affiliations:** 1 The University of Chicago, Chicago, Illinois, United States; 2 Western Michigan University, Kalamazoo, Michigan, United States; 3 University of Chicago, Chicago, Illinois, United States

## Abstract

COVID-19 poses unique challenges to family caregivers. This study explores how family caregivers for older adults with cognitive impairments experience and make decisions about caregiving during a global pandemic. Using purposive sampling, 63 family caregivers across eight states participated in open-ended qualitative interviews (2019-2020), until thematic saturation was reached. Questions broadly examined caregivers’ experiences and decisions, focusing on decisions made around type of care setting. Questions about responses to the Pandemic were added as events unfolded. States were selected to represent variation in Home and Community Based Service (HCBS) expenditures as a percentage of total Medicaid long-term services and supports expenditures. Family caregivers experienced significant concern about COVID-19 itself, and about the indirect consequences of caregiving caused by the pandemic. Caregivers also displayed flexibility and adaptability in ceasing selected services, contingently continuing services, and utilizing telemedicine and other remote healthcare interventions to protect their loved ones. Many family caregivers utilized remote health care tools such telemedicine, no-contact prescription and grocery delivery. Such measures improved service access and reduced caregiver workload. Given the persistent challenges posed by COVID-19, long-term service organizations have an opportunity to enhance their policies to meet the needs of caregivers and those they care for. There is a need to expand telemedicine and other remote healthcare tools, while adapting these technologies to the needs of families. Also, procedures are needed for safe pathways to utilize HCBS and nursing care during a pandemic including communication supports, sufficient PPE, increased staffing, and utilization of evidence-based protocols.

